# A New Pathy

**Published:** 1857-09

**Authors:** J. S. Thomas

**Affiliations:** Bernadotte, Ill.


*A New Pathy.*

BERNADOTTE, ILL., April 17, 1857.

PROF. N. S. DAVIS:—Another new "Pathy" is about to dawn upon the medical world. Shades of infinitessimal pills, red-pepper and steam, where are your virtues now? Come, quacks, here is a recipe for you.

An old woman in this town, on last Sabbath, while the citizens were attending Divine service, under the broad glare of the noonday sun, exhumed the remains of her son, who died some three years ago of pulmonary consumption, for the purpose of procuring a portion of its lungs to "make a tea" to cure a sick member of her family.

As there was nothing but bones in the coffin, of course the patient did not get its delectable "tea." I did not hear how this expectorant, this "balsam for the lungs," was to be prepared, but will let the Grave-o-pathists settle it for themselves.

It does not seem possible that we have, in this enlightened noonday of the nineteenth century, such awfully ignorant people; but it is nevertheless so, for this act can be attested by the best citizens in the place.

Yours truly, J. S. THOMAS.

